# Supplementary material for: Aluminum-Doped Zinc Oxide Improved by Silver Nanowires for Flexible, Semitransparent and Conductive Electrodes on Textile with High Temperature Stability
Source: Materials (Basel). 2023 May 25;16(11):3961. doi: 10.3390/ma16113961 (PMC10253794; doi:10.3390/ma16113961)
Supplement: Supplementary file 1 [file materials-16-03961-s001.zip › materials-2385659-supplementary.pdf]

Aluminum-Doped Zinc Oxide Improved by Silver Nanowires for Flexible, Semitransparent and Conductive Electrodes on Textile with High Temperature Stability

Maximilian Lutz Hupfer, Annett Gawlik, Jan Dellith and Jonathan Plentz *

Leibniz Institute of Photonic Technology (IPHT), Albert-Einstein-Str. 9, 07745 Jena, Germany;
maximilian.hupfer@leibniz-ipht.de (M.L.H.); jan.dellith@leibniz-ipht.de (J.D.)
* Correspondence: jonathan.plentz@leibniz-ipht.de

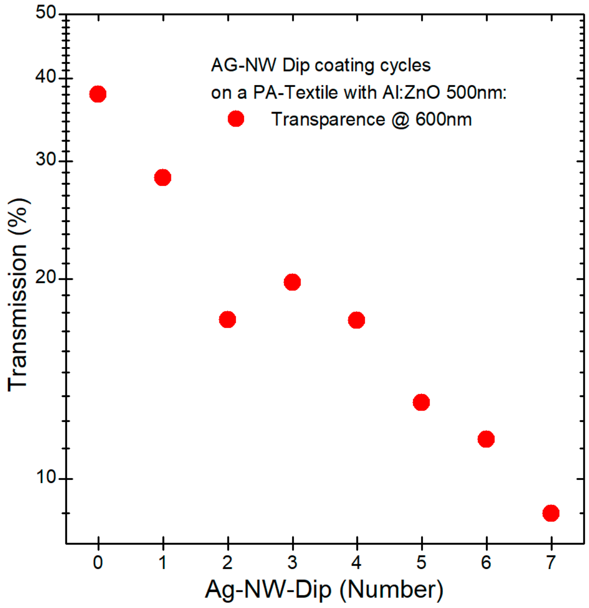

Figure S1: Transmission @ 600nm of a polyamide-based calendered textile as a function of the immersion in an Ag-NW dispersion (2mg/ml in EtOH, 30s per dip) with (red) an additional Al:ZnO (500nm @180°C) coating.

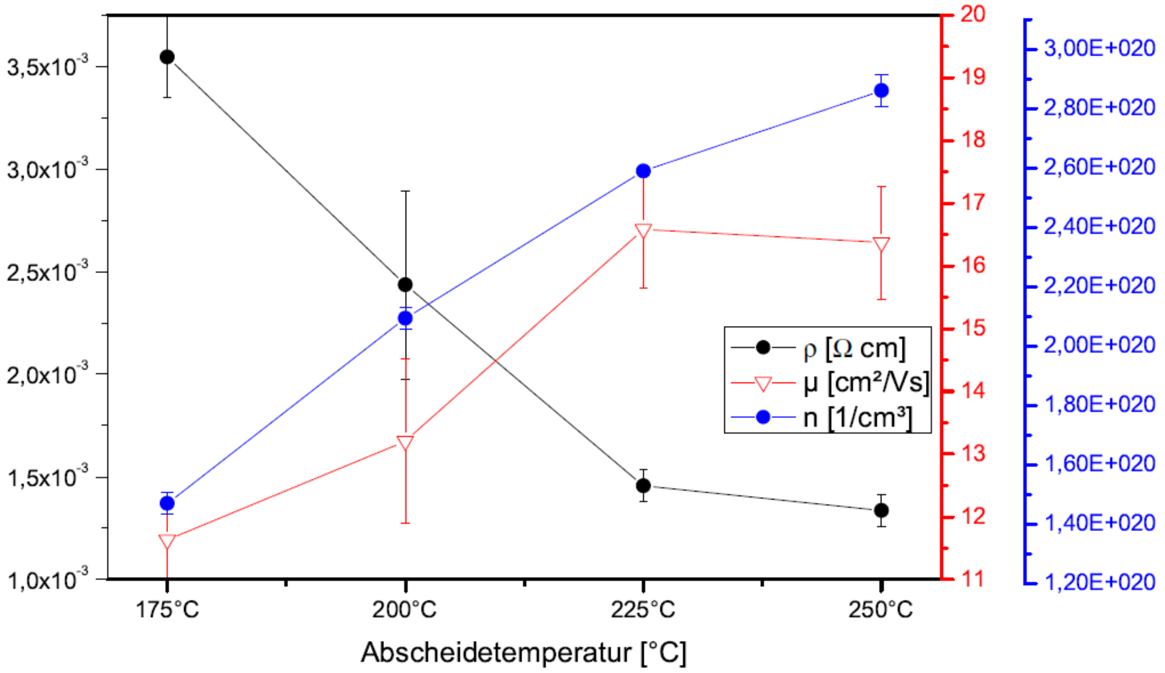

Figure S2: Charge carrier density n, mobility μ and resistivity for Al:ZnO films with different deposition temperature, a target film thickness of 400 nm and N=23:1.

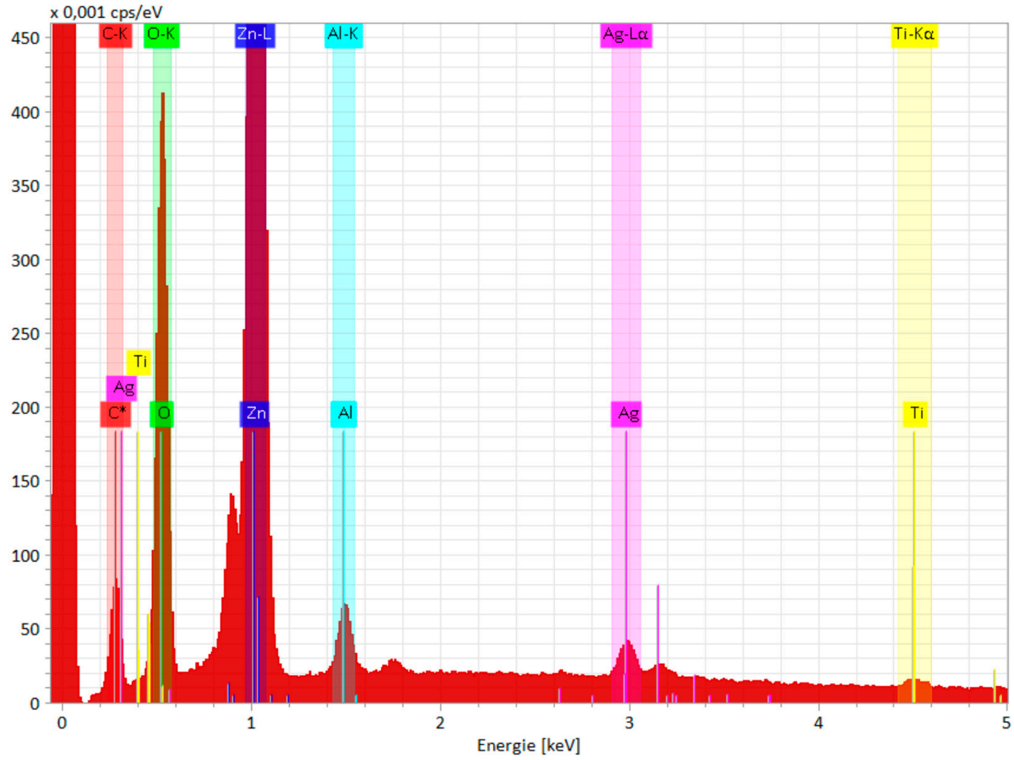

Figure S3: EDX element analysis of polyamide-based calendered textile coated with Al:ZnO and combined with 4 dips of Ag-NW
